# Spatio-temporal photonic metalattice

**DOI:** 10.1038/s41377-026-02452-w

**Published:** 2026-10-29

**Authors:** Sven Stengel, Wallace Jaffray, Farhan Ali, Mustafa Goksu Ozlu, Colton B. Fruhling, Maria Antonietta Vincenti, Domenico De Ceglia, Michael Scalora, Alexandra Boltasseva, Vladimir M. Shalaev, Andrea Di Falco, Marcello Ferrera

**Affiliations:** 1 Institute of Photonics and Quantum Sciences, Heriot-Watt University, Edinburgh, EH14 4AS UK; 2 School of Physics and Astronomy, University of St. Andrews, St Andrews, UK; 3 Elmore Family School of Electrical Computer Engineering and Birck Nanotechnology Center, Purdue University, West Lafayette, IN USA; 4 Department of Information Engineering, University of Brescia, Brescia, Italy; 5 CNR—National Institute of Optics, Via Branze 45, 25123 Brescia, Italy

**Keywords:** Optical materials and structures, Applied optics

## Abstract

When coherent light interacts with an ordered lattice with periodicity comparable to its wavelength, a diffraction pattern emerges. The same principle applies in crystallography, where X-rays are employed to reveal atomic structures. By asking ‘when’ the diffractive object exists, rather than ’where’, we implicitly introduce time as a design parameter, thus enabling the creation of spatio-temporal metalattices. The temporal modulation of optical properties complements the spatial one, enabling functionalities such as dynamic reconfigurability, nonreciprocal behavior, coherent amplification, and tailored spectral response. However, for these effects to be relevant an extreme temporal gradient of the refractive index is necessary. In the present work, we realize a two-dimensional spatio-temporal metalattice by integrating a physically patterned spatial modulation with an orthogonal temporal lattice induced by interfering ultrafast pulses, using highly nonlinear transparent conducting films operated within their low-index window. While the optical pumps experience a uniform medium, the spatio-temporal lattice can be probed by either an internally generated third harmonic signal, or an externally supplied probe wave. The transient lattice shows comparable diffraction efficiency to the equivalent physical structure and is also dynamically reconfigurable via a broad range of parameters, including the pulses delay, incidence angle, and wavelength. System flexibility is also demonstrated through phase-mask wavefront modulation, highlighting exceptional versatility for ultrafast transient lithography and photon manipulation in both momentum and frequency. This approach shifts device design from fixed fabrication constraints to radiation engineering, opening new pathways towards ultrafast reconfigurable photonics.

## Introduction

Transparent conductors, which are widely recognized in industry for their electro-optic roles in photovoltaic and touch screen technologies^1^, have recently been investigated for their nonlinear optical responses within spectral regions where their refractive index falls well below unity^2–4^. In this regime, under ultrafast optical excitation, an unprecedented temporal refractive index gradient has been observed. This phenomenon underpins several remarkable experimental outcomes, including unitary refractive index modulation^5^, broadband spectral routing of ultrafast pulses^6^, dynamic manipulation of photon energy and momentum^7–9^, and a generalized form of Snell’s law for non-reciprocal metasurfaces where a temporal phase gradient complements the conventional spatial gradient^10,11^. In addition, the unprecedented speed of the material response, capable of tracking few-cycle optical excitations^12,13^, has sparked renewed interest in exploring optical phenomena within time-varying systems. Particular attention has been directed towards emerging concepts such as photonic time crystals^14–20^ and ultra-efficient coherent amplification^21^. This is well justified by the opportunity to finally overcome the longstanding trade-off between amplitude and speed in nonlinear processes^22^. With time now effectively incorporated into the engineering toolbox for designing advanced optical materials and metamaterials, a new frontier has emerged: the development of systems capable of ultrafast manipulation of optical radiation across the classic and quantum regimes^8,23,24^. Motivated by this paradigm shift, we investigate diffraction from optically induced lattices in low-index media and directly compare their scattering response with that of conventional fabricated nanostructures.

In this context, aside from permanently photo-imprinted structures^25^, optically induced transient gratings have long established themselves as a powerful tool for studying light-matter interaction and material dynamics^26–28^. Since their early demonstrations in non-solid-state systems, including molecular and plasma-based media^26,29^, transient gratings have been generated by interfering phase-coherent optical beams to produce a spatial modulation of the refractive index capable of diffracting an incident probe. Over the years, this concept has progressively evolved towards solid-state implementations, where transient gratings have been extensively employed for spectroscopic investigations of electronic, vibrational, thermal, and acoustic processes in condensed matter systems^30–32^. These approaches have enabled the study of carrier transport, molecular relaxation, acoustic propagation, and nanoscale dynamics in a broad range of materials^27,28,31^. More recently, transient gratings induced in solid-state platforms such as vanadium dioxide (VO_2_) have also been explored, including the excitation of guided-mode resonances and dynamically reconfigurable optical responses^33,34^. A different class of dynamically reconfigurable systems has also emerged through externally driven tuning mechanisms, including electro-optic, acousto-optic, and phase-transition-based platforms^31,33,35–37^.

Despite their diversity, previously listed transient-grating approaches operate effectively in an ON/OFF regime, where the transient structure acts as a switchable diffraction object. In these systems, the key requirement is the existence of a spatial refractive-index modulation capable of diffracting light, while the temporal refractive-index gradient is not large enough to become an active mechanism: in some cases the modulation may be ultrafast but shallow, while in others the index change may be stronger but too slow. As a result, the optical field interacts with the transient lattice in an effectively quasi-static manner, where diffraction arises from the presence or absence of the grating rather than from the temporal evolution of the medium. Consequently, although “transient” these systems are not “time-varying” and they cannot access the regime required for simultaneous manipulation of both photon momentum and photon energy through time-dependent interaction^38^.

Here, we demonstrate a strongly time-varying photonic lattice operating in a regime enabled by the giant ultrafast nonlinearities of low-index transparent conducting oxides. In contrast to conventional transient gratings, the temporal evolution of the refractive index itself becomes the active optical mechanism. The system therefore operates beyond a simple ON/OFF diffraction picture, enabling simultaneous control over both momentum and energy of light.

To achieve this, we unify two distinct concepts into a single optical platform: a horizontally aligned ridge grating patterned onto an aluminum zinc oxide (AZO) film, and a vertically oriented optically induced lattice generated by ultrafast near-infrared beam interference. The resulting spatio-temporal structure remains almost “invisible” to the longer-wavelength pump radiation because the grating periodicity is larger than the effective pump wavelength, yet it can be directly interrogated either through an internally generated and locally enhanced third-harmonic (TH) signal or by means of an externally supplied probe. The resulting diffraction pattern directly reveals the underlying spatio-temporal lattice responsible for its formation, the same way x-ray diffraction exposes atomic arrangements in crystals. Beyond conventional multi-beam interference schemes, we further demonstrate complex diffraction landscapes generated through the use of phase masks, thereby extending the platform towards nonlinear single-beam ultrafast wavefront shaping.

The present platform introduces several key advances over previously reported results: (i) the demonstrated structure operates as a genuinely time-varying photonic lattice rather than a conventional transient ON/OFF grating, enabling simultaneous control over both photon momentum and photon energy through giant ultrafast temporal refractive-index gradients^38^; (ii) the proposed architecture provides a highly versatile platform for ultrafast reconfigurable photonics, supporting both internally generated and externally supplied probing together with real-time control through wavelength, intensity, incidence angle, polarization, temporal delay, and spatial wavefront engineering^39^; (iii) the work provides the first direct demonstration that an optically induced and fully reversible structure can reproduce scattering properties comparable to those of a physically fabricated equivalent. In other words, we effectively “fool” photons into interacting with a transient optical illusion as if it were a real nanostructure^38^.

The results reported in the present manuscript are readily adaptable to a wide spectrum of advanced applications, including dynamic metasurface design, high resolution spectral analysis, ultrafast wavelength and spatial division multiplexing, on-chip engineering of high-harmonic generation, and ultrafast transient photo-lithography. Moreover, many of these directions point towards potential relevance in the quantum domain, where analogous principles may inform or enable emerging quantum photonic architecture and use-cases^40^.

## Results

### Diffraction from a spatio-temporal metalattice

**Fig. 1:**
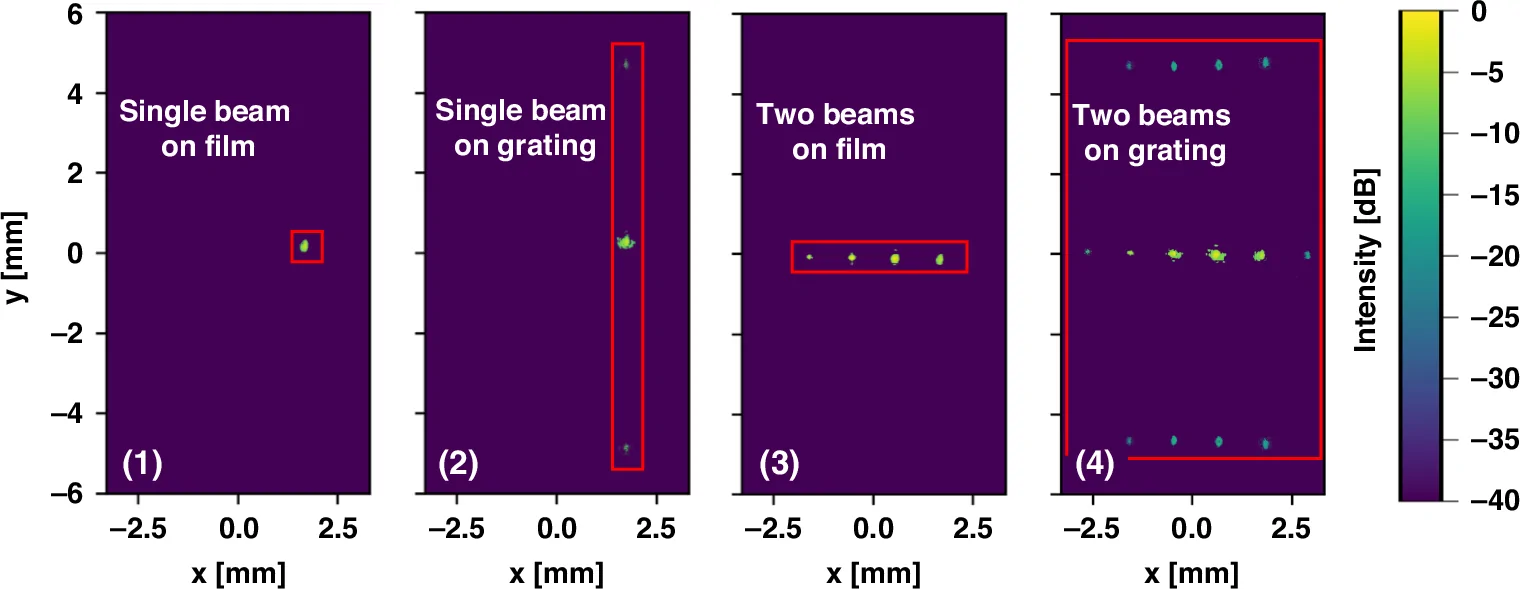
**Spatio-Temporal diffraction.** Experimentally recorded TH output intensity patterns captured via a visible CCD camera for four excitation configurations (*θ*_i_ =  6^∘^): (1) Single beam on unpatterned AZO film; (2) Single beam on meta-grating; (3) two beams on unpatterned film; (4) two beams on meta-grating. Panel (1) shows standard THG from the plain AZO surface. Panels (2) and (3) isolate the diffraction effects arising from the structural and temporal gratings, respectively. Panel (4) reveals the full spatio-spectral interference pattern resulting from the combined static and transient lattices

To characterize the scattering properties of the proposed spatio-temporal metalattice, we first investigated the third-harmonic emission generated under four excitation configurations combining single- and double-beam illumination with either an unpatterned AZO film or a fabricated metagrating. The resulting far-field diffraction patterns, shown in Fig. 1, reveal the individual and combined signatures of the structural and optically induced gratings (which are set to be orthogonal one to another), providing the experimental foundation for the analysis that follows. The experimental arrangement, together with the sample geometry and fabrication details, is described in the “Materials and methods” section.

The spatial distributions of TH signals for all four used configurations are shown in Fig. 1 for an input angle *θ*_i_ = 6^∘^. More specifically, in panel (1) a single pump beam, incident on the unpatterned film, generates a collinear TH. In panel (2) a single pump incident on the metagrating produces two additional TH beams symmetrically above and below the central TH signal. The associated vertical emission angles are consistent with standard grating theory applied to the TH signal. Panel (3) shows the result of two beams incident on the unpatterned film, yielding four spatially separated TH spots whose origin will be explained in detail in the following paragraphs. Finally, panel (4) displays the full spatio-temporal interference pattern produced by two pump beams incident on the metagrating, which leads to fourteen distinct TH spots in the far field, revealing the embedded two-dimensional lattice produced by the combined static and transient structures.

Special attention must be given to the TH pattern recorded in the third panel of Fig. 1, where the observed TH signals can be attributed to two fundamental mechanisms: (i) photon momentum (wavevector) conservation pertaining to the TH process^13^; (ii) far field interference due to the transient temporal grating formed by the two pump beams. These two contributions are now examined in detail.

**Fig. 2:**
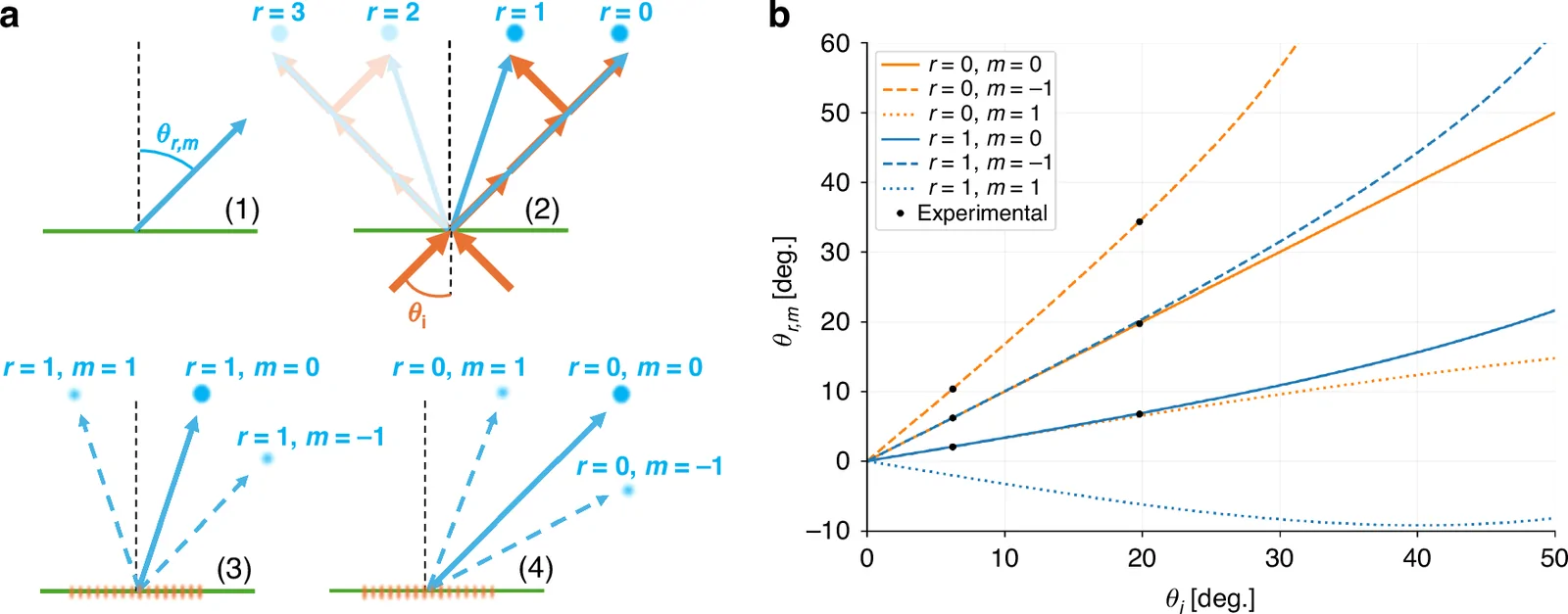
**Third harmonic angular analysis (two beams configuration).** **a** Schematic of the mechanism determining the horizontal angular distribution of TH emission. Panels (1) and (2) show the phase matching geometry for fundamental TH beams produced by two-beam interaction inside the AZO films where *θ*_*r*,*m*_ indicates the output angle between a given TH ray and the surface normal. Panels (3) and (4) illustrate how each phase matched TH beam (indexed by *r*) is subsequently diffracted by the temporal grating; only the first diffraction orders are shown for clarity (indices m =  ± 1). **b** Calculated TH emission angle *θ*_*r*,*m*_ for all considered beams (r = 0, m = {0, 1, -1} and r = 1, m = {0, 1, -1}) is plotted as a function of the pump incidence angle *θ*_i_. The curves demonstrate that for pump incidence below 30^∘^, several diffracted TH rays nearly coincide with the phase-matched emission directions, producing strong overlap between diffraction and intrinsic THG channels. NOTE: Along the manuscript “horizontal” and “vertical” refer to the experimental optical table

Regarding point (i), the THG process involves the mixing of three pump photons, yielding four distinct k-vectors according to the vector diagram in Fig. 2a-1 and Fig. 2a-2, which are aligned with the four TH spots recorded in Fig. 1, 3rd panel. Two of these TH k-vectors arise from collinear summations of fundamental photon wavevectors, resulting in TH emission along the pump propagation axis. The remaining two TH k-vectors at smaller angles, correspond to the non-collinear phase-matching configurations.

In this regime, it is important to clarify the role of phase matching. In conventional bulk nonlinear optics, phase matching is required to ensure constructive buildup of the nonlinear signal over extended interaction lengths, where even small phase mismatches accumulate and significantly reduce conversion efficiency. In contrast, the present system operates over a deeply subwavelength thickness (on the order of a few hundred nanometers), such that no significant longitudinal phase accumulation occurs during the interaction. As a result, the conventional phase-matching condition does not apply in the usual sense. The nonlinear emission is instead governed by local field interactions, and the only relevant constraint is momentum conservation at the generation point, which fully determines the allowed emission directions, as described in Fig. 2. This relaxation of phase-matching requirements is a well-known and advantageous feature of subwavelength nonlinear platforms. A vectorial analysis based on photon momentum conservation enables the calculation of the exact TH emission angles (*θ*_*l*,*r*_) using the following expression:

1 $${\theta }_{l,r}({\theta }_{{{\rm{i}}}})={\tan }^{-1}\left(\frac{l-r}{l+r}\tan ({\theta }_{{{\rm{i}}}})\right)$$

Here, *θ*_i_ denotes the angle of incidence of the fundamental beams, and *l* and *r* represent the number of photons “donated” from the left and right incident beams, respectively (see Supplementary Information for further discussion). Only combinations satisfying the condition *l* + *r* = 3, yield valid TH emission angles, consistent with energy conservation. Each configuration corresponds to a distinct phase-matching pathway, allowing a precise prediction of the TH emission direction.

**Fig. 3:**
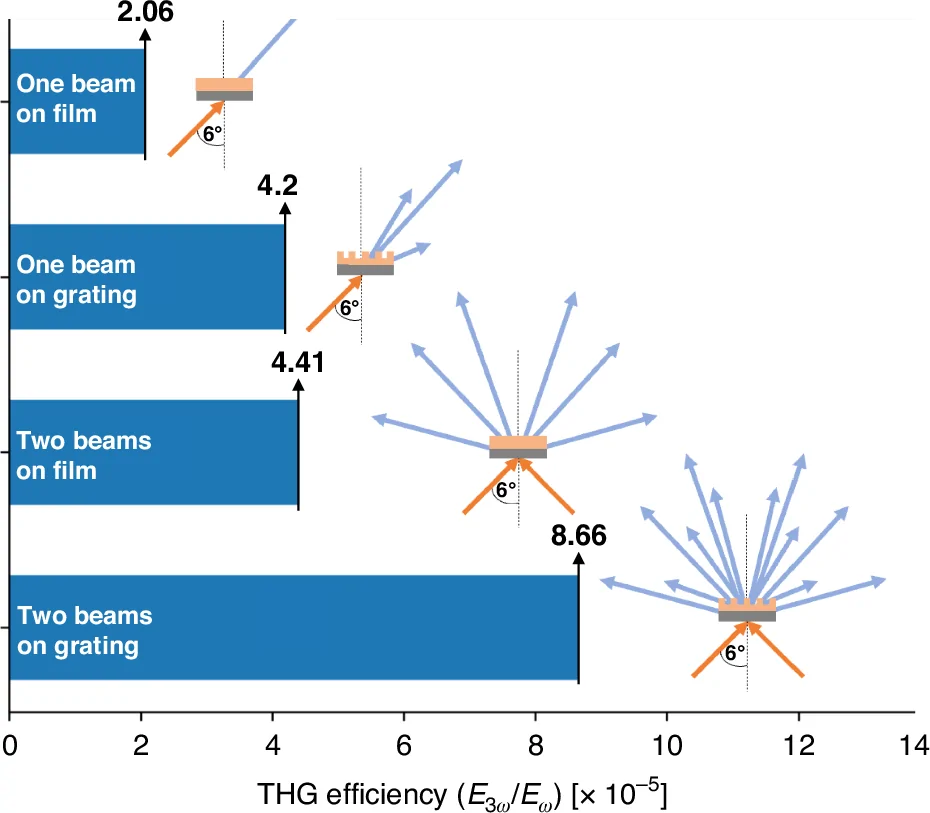
**Third harmonic generation efficiency.** From top to bottom, the total transmitted THG efficiencies are reported for: (i) single beam on unpatterned film; (ii) single beam on meta-grating; (iii) two beams on unpatterned film; (iv) two beams on meta-grating. Efficiency calculations include all detectable diffracted THG orders and all incoming fundamental energy (in the two-beam configuration the total energy of both beams is considered so the input energy is twice the case of the single beam)

**Fig. 4:**
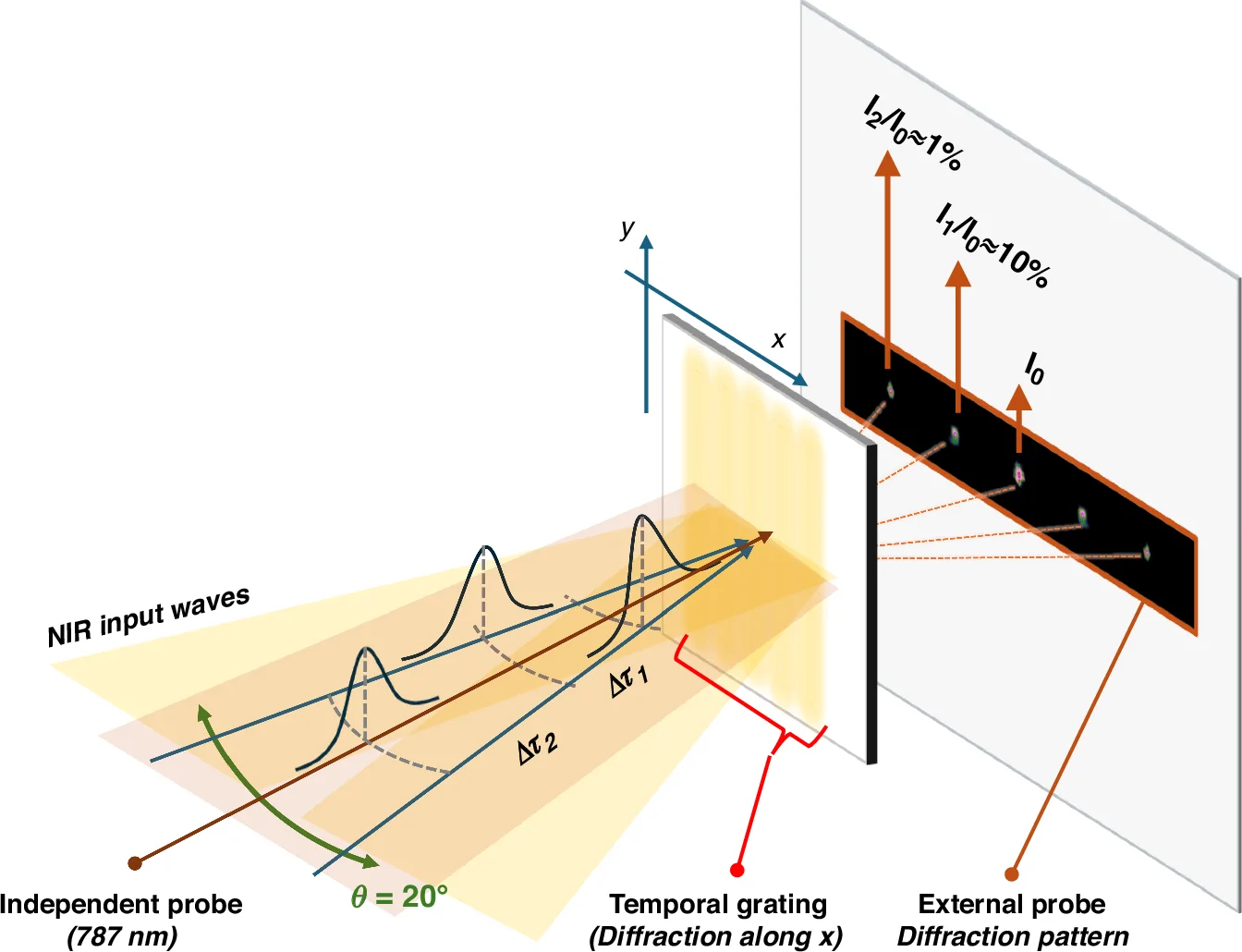
**Independent probe diffraction from an optically induced temporal grating.** Schematic of the pump-probe configuration in which a transient grating is generated by two interfering near-infrared (NIR) pump beams, while an independent probe beam (*λ* = 787 nm) is used to interrogate the induced refractive index modulation. The pumps are operated below the TH generation threshold to ensure that the detected signal arises solely from diffraction of the probe. The transient grating, oriented along the *x*-direction, redistributes the incident probe into well-defined diffraction orders, which are directly recorded on the detection plane

With regards to point (ii), the combined effect of two-beam interference and pronounced nonlinearities in low-index media gives rise to a transient grating oriented perpendicular to the structural metagrating (see sample schematic in Materials and methods). This optically induced periodic structure also produces its own TH interference pattern in the horizontal direction, as illustrated in Fig. 2a-3 and Fig. 2a-4. These interference orders are labeled by indices *m*. As previously done for the emission angle due to photon momentum conservation, a simplified angular analysis can also be applied to the interference orders arising from the transient temporal grating. By applying the standard grating equation to the generated TH light, we obtain the following expression for the TH angle (*θ*_*r*,*m*_), where we have dropped the redundant *l* index which can be recovered from energy conservation:

2 $${\theta }_{r,m}={\sin }^{-1}\left(\frac{m\lambda }{\Lambda }-\sin \left({\theta }_{r}\right)\right)$$

Here, *m* denotes the interference order, *Λ* is the groove spacing, *λ* is the third-harmonic wavelength which is equal to *λ* = *λ*_i_/3 (*λ*_i_ being the incident pump wavelength), and *θ*_*r*,*m*_ is the TH propagation angle as defined by Eq. (1). The period of the transient grating produced by the interference of the two pump beams is therefore:

3 $$\Lambda =\frac{{\lambda }_{{{\rm{i}}}}}{2\sin \left({\theta }_{{{\rm{i}}}}\right)}\,=\,\frac{3\lambda }{2\sin \left({\theta }_{{{\rm{i}}}}\right)}$$

If now we focus solely on the collinear TH labeled *r* = 0, for which *θ*_*r*_ = *θ*_i_, and substituting Eq. (3) into Eq. (2), we obtain the following formula linking the angle of the diffraction order with the incident angle:

4 $${\theta }_{r,m}={\sin }^{-1}\left(\left(\frac{2m}{3}-1\right)\sin \left({\theta }_{{{\rm{i}}}}\right)\right)$$

Evaluating *θ*_*r*,*m*_ from Eq. 1 and Eq. 4 for the two experimental incidence angles (i.e., 6^∘^ and 20^∘^) reveals near-perfect angular coincidence between the phase-matched and diffracted TH beams. Fig. 2b shows a complete view of the output angle *θ*_*r*,*m*_ as a function of the input angle *θ*_i_ for all fundamental TH phase-matched beams (*m* = 0) and their first interference orders (*m* = ± 1).

We now turn back to Fig. 1, where in the 4th panel the most revealing feature is the central horizontal band, where six TH signals are recorded in the far field. The four central spots arise from a combination of nonlinear phase matching and temporal grating interference, as discussed above. The two outermost beams along the horizontal line, observed at the largest angles, cannot be accounted for by Eq. 1 or by higher-order wave-mixing. Therefore, we attribute their origin exclusively to temporal-grating interference (see Supplementary Information for details). Notably, the temporal *r* = 0, *m* = − 1 interference order exhibits an intensity comparable to its spatial counterpart, indicating that the transient grating mirrors the influence of a physical grating.

When comparing panels 3 and 4 in Fig. 1, the absence of the outermost TH spots in the former (*r* = 0, *m* = − 1 and mirrored interference order) is easily explained by a robust generation enhancement induced by the metagrating which allows those interference orders to exceed the detection threshold for the two-beam-on-grating configuration.

To expand on this last point, a detailed analysis of the TH generation capabilities of our system is shown in Fig. 3 where experimentally measured TH efficiencies, are reported for all four experimental configurations. Here, conversion efficiency is defined as the ratio of the forwardly emitted TH energy normalized to the incident fundamental energy (for the two-beam case, the total incident energy of both pumps is used). The measurements show a pronounced efficiency increase when the grating is present, which we attribute to some local field enhancement (see Supplementary Information).

To investigate the optimization of THG as a function of both wavelength and grating geometry we performed first, a wavelength scan of a single pump beam on the grating, followed by numerical simulations on both filling fraction and grating pitch. These results are reported in detail in the Supplementary Information and they show that the main enhancement comes from operating close to the crossover wavelength, while only an additional increment in the THG efficiency can be attained by optimizing the grating design.

When evaluating overall system performance, it is also important to clarify the notion of diffraction efficiency for the temporal grating. In the linear regime, the diffraction efficiency of order *m* for any standard grating is typically defined as *η*_*m*_ = *P*_*m*_/*P*_inc_, where *P*_*m*_ is the power in the *m*-th diffracted order and *P*_inc_ is the incident power at the same frequency. This definition, however, is not applicable to the internally generated TH signal considered here. In fact, at the pump wavelength, the transient lattice is effectively subwavelength and no far-field scattering occurs. As a result, the incident pump power cannot serve as a meaningful reference for the diffraction efficiency.

Given that at the TH frequency the system is more appropriately described as a spatially periodic distribution of nonlinear emitters rather than passive scatterers, a meaningful alternative parameter is the power redistribution of the generated TH signal. This analysis can be evaluated looking at the fraction of emitted power between adjacent interference orders or by comparing each interference order emission with the one of an unpatterned film under identical excitation conditions. Following these alternative metrics, the response of our system is quite remarkable with an efficiency exceeding 200%, given that the patterned grating produces a stronger TH output when compared to the bare film, despite the reduced active volume.

### Independent-probe diffraction: isolating phase-matching effects

To further underline the flexibility of our system and to unambiguously disentangle phase-matching contributions from pure diffraction, we performed an additional set of experiments using an independent external probe. In the original configuration, the detected TH signal is generated within the material and therefore simultaneously carries signatures of both nonlinear phase-matching pathways and interference patterns from the transient grating. As a result, the angular distribution of the emitted signal reflects a superposition of intrinsic THG momentum conservation and grating-induced scattering, making it non-trivial to isolate the contribution of each mechanism.

To address this point without downsizing the scope of the work, we implemented a pump-probe configuration in which the transient grating is generated by two interfering near-infrared (NIR) pump beams operated below the third-harmonic generation threshold, while a separate probe beam (*λ* = 787 nm) was used to interrogate the induced refractive index modulation. In this configuration, the detected signal originates exclusively from diffraction of the probe by the optically induced lattice, with no contribution from internally generated nonlinear emission. A schematic of the experimental settings, together with the associated resulting diffraction pattern, are reported in Fig. 4. These measurements confirm that the optically induced refractive index modulation effectively redistributes an external probe into well-defined diffraction orders, thereby directly demonstrating the diffractive capability of the temporal lattice independently of nonlinear wave-mixing processes.

At this point, we should mention that, in this configuration, a conventional definition of scattering efficiency applies, as the incident probe field is directly redistributed among different angular channels by the transient grating. In principle, the diffraction efficiency of order *m* can be defined as *η*_*m*_ = *P*_*m*_/*P*_inc_, where *P*_*m*_ is the power in the *m*-th diffracted order and *P*_inc_ is the incident probe power. However, experimental conditions did not allow for full and accurate calibration of all diffracted channels relative to the incident beam, and therefore a strict evaluation of *η*_*m*_ could be reliably extracted.

**Fig. 5:**
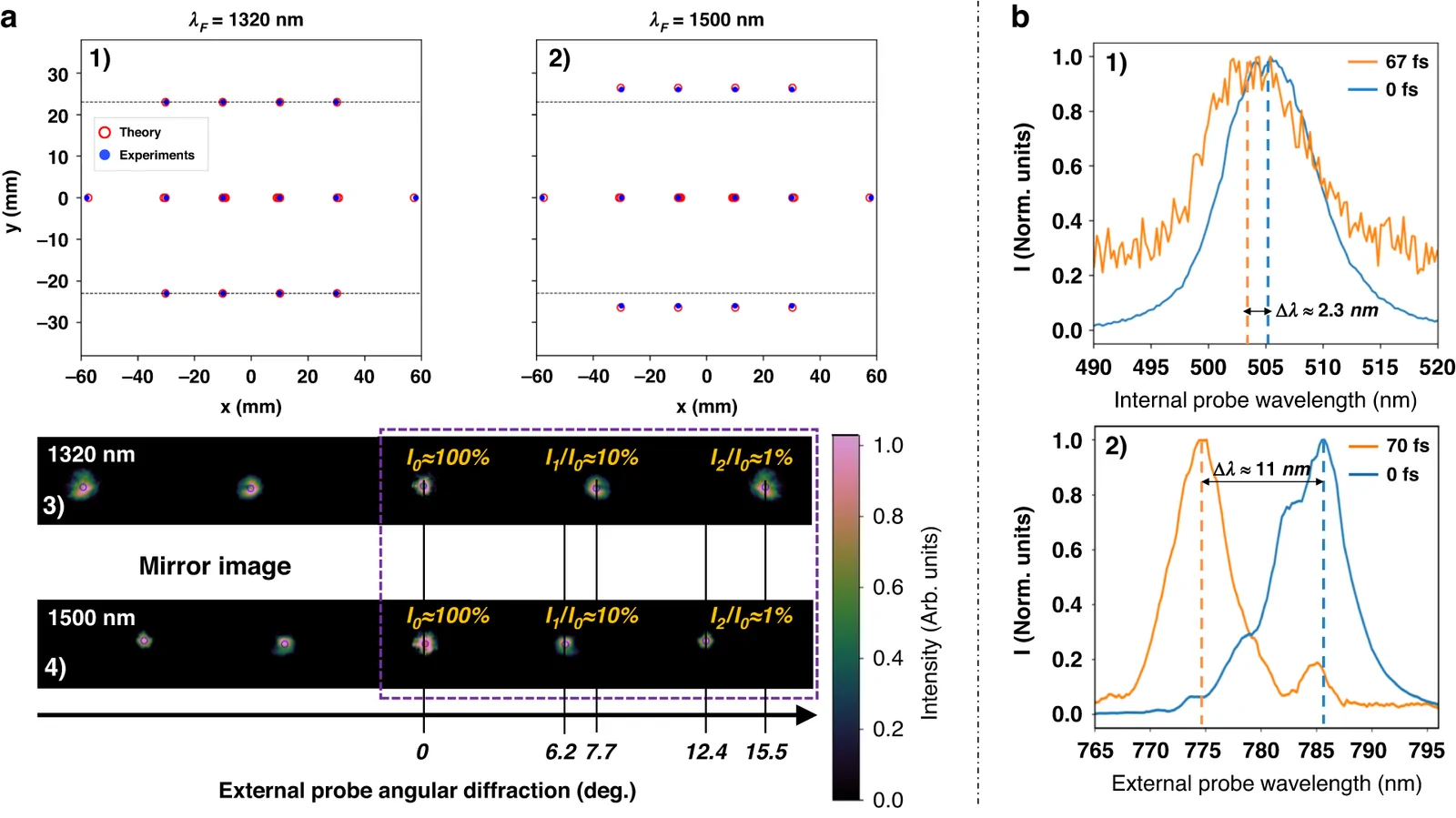
**Metagrating tunability via wavelength and temporal delay.** **a** *Wavelength tunability*. Internal probe (two pumps on metagrating): experimental (blue dots) and theoretical (red circles) positions of the third-harmonic (TH) diffraction orders for pump wavelengths of 1320 nm (panel 1) and 1500 nm (panel 2) at an incidence angle of 20^∘^. External probe (two pumps on unpatterned film): representative diffraction patterns recorded for the same pump wavelengths (*λ*_P_ = 1320 nm, panel 3, and *λ*_P_ = 1500 nm, panel 4). **b** *Temporal tunability*. Internal probe: experimentally measured TH spectra for two pump-pump delays that maximise the frequency shift (panel 1). External probe: intensity spectra for two probe-pump delays (between the synchronized pumps and the independent probe) that maximise the spectral shift (panel 2). The use of an external probe enables decoupled control of lattice formation and probing, resulting in significantly enhanced spectral tunability. NOTE: Only the spots within the magenta dashed box were directly recorded by the VIS camera owing to its limited field of view; the remaining spots are shown as mirror images to reproduce the full symmetric diffraction pattern observed experimentally

What can be robustly determined is the relative distribution of power among the diffraction orders. In particular, the intensity ratios between the diffracted orders and the zero-order beam, reported in Fig. 4 and Fig. 5a (i.e., *I*_1_/*I*_0_ and *I*_2_/*I*_0_), provide a direct and quantitative measure of the redistribution induced by the transient grating. These results clearly demonstrate that the externally applied probe is efficiently redistributed into well-defined off-axis channels, confirming the strong diffractive behavior and effectiveness of the optically induced structure.

### Lattice tunability as a function of wavelength and time delay

In the following section, we are going to assess the tunability of the spatio-temporal lattice with respect to both wavelength and temporal delay. For this purpose, we will figure both configurations with internally generated and externally supplied probes. For the case of an internally generated TH probe, the transient grating pitch can be proven to be wavelength independent given the frequency relation between pump and probe (see Eq. 4).

We validate this experimentally by tuning the pump wavelength and recording the resulting diffraction patterns reported in Fig. 5a1-2. The data confirm that the interference pattern from the temporal grating remains fixed in angle when the wavelength changes. Conversely, the interference pattern from the static spatial grating shifts vertically (dashed lines in Fig. 5a1-2 highlight this motion). Measured beam positions (blue dots) match the theoretical predictions red circles computed from Eq. (1) and Eq. (4) with excellent agreement.

For the case of externally supplied probe, Fig. 5a-3 and Fig. 5a-4 report the experimentally recorded one-dimensional diffraction patterns at 787 nm for two different pump wavelengths (1320 nm and 1500 nm). In this case, pumps and probe are not linked via any frequency conversion process, and by varying the pump wavelength, the pitch of the induced lattice is modified, leading to a corresponding shift in the angular distribution of the diffracted probe. In conclusion, while the internal probe configuration has the advantage of not requiring an additional external probe beam, the use of an independent probe enables a fully decoupled platform in which the lattice properties and the probing wavelength can be independently set.

So far, we have explored our spatio-temporal metasurface by varying wavelength and incidence angle. However, because the system is intrinsically time-dependent, time itself can be used as one of the control parameters. As before we will look into both configuration with internal and external probe. For the former case we first measured the device response as a function of the delay between the two trains of pump pulses. Fig. 5b-1 shows spectral measurements of the generated TH signals recorded at an incidence angle of 6^∘^ and for two representative time delays (*τ* = 67 and *τ* = 0 fs) for which the frequency shift is maximised. These data show a moderate tuning capability of the pulse carrier of about 20% of its bandwidth.

A complementary picture emerges when the temporal lattice is interrogated using an external probe. In this configuration, the delay is introduced between the independent probe and the two synchronized pump pulses that generate the grating. By varying this probe-pump delay, the probe samples different stages of the ultrafast refractive-index evolution, resulting in a pronounced shift of its output spectrum. This behavior is illustrated in Fig. 5b-2, where two representative spectra show a frequency displacement significantly larger than that observed in the internally generated probe scheme. In particular, the shift reaches approximately 110% of the probe bandwidth, compared with the  ~ 20% tuning obtained in the previous configuration. The delay-controlled tuning observed in both investigated cases, stems from a time-refraction mechanism that, under specific conditions, can lead to spectral shifts up to 40 nm as previously demonstrated^41^.

### Single beam wavefront modulation for complex diffraction patterns

At this stage, it is natural to ask whether temporal patterning complexity can extend well beyond the simple two beam interference case. While the specific geometry of the induced lattice is not central to the underlying mechanism, the ability to generate more complex wavefronts provides a direct demonstration of our systems flexibility. In general, arbitrary intensity distributions can be produced using phase masks, diffractive optical elements, or spatial light modulators, all relying on the same principle of wavefront engineering.

**Fig. 6:**
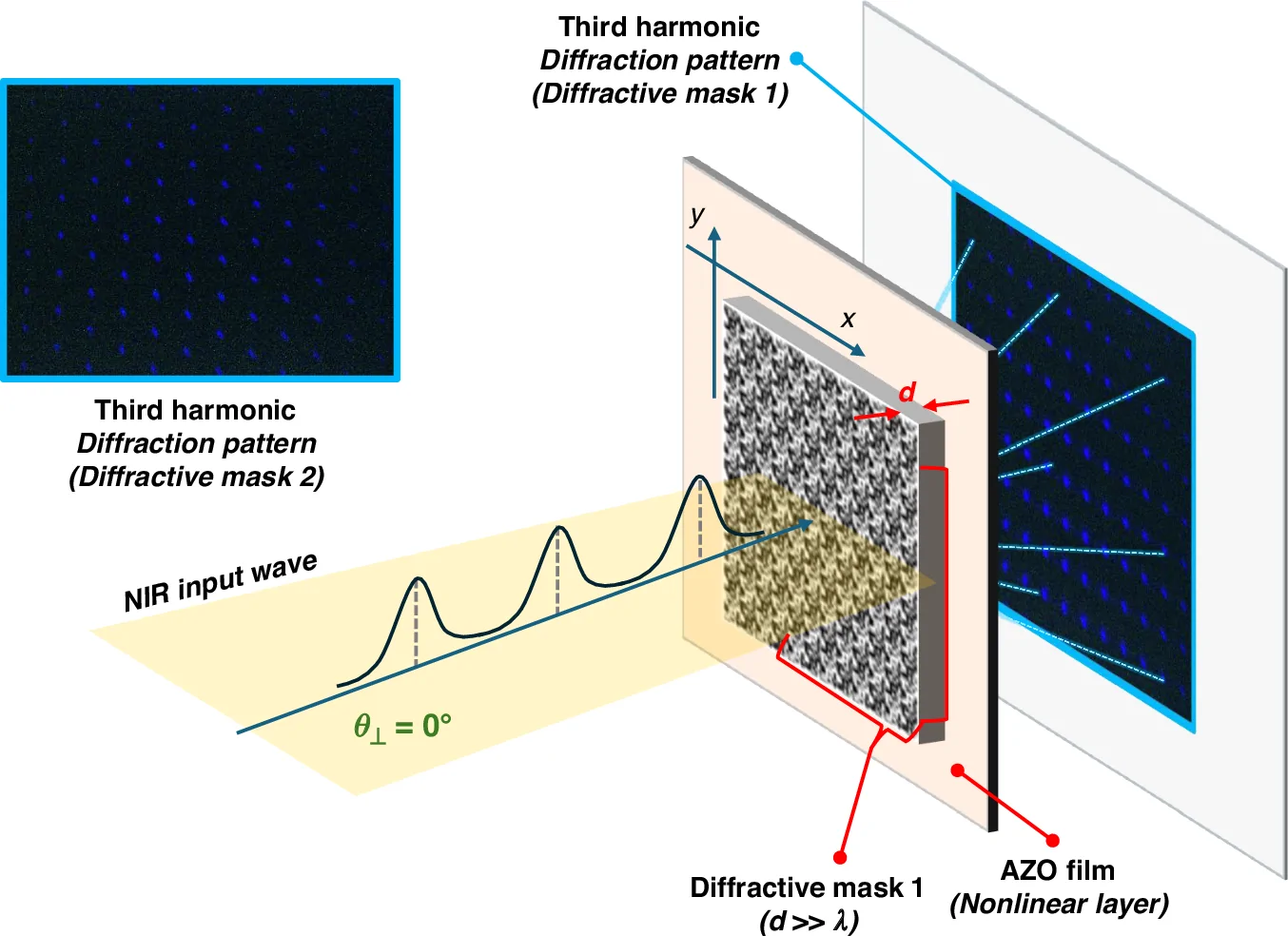
**Single-beam generation of complex diffraction patterns via wavefront shaping.** Schematic of the experimental configuration in which a NIR pump beam (*λ* = 1320 nm) is spatially modulated by a diffractive mask before reaching the AZO nonlinear film in the far-field (*d* ≫ *λ*). The mask is designed to generate a prescribed two-dimensional intensity distribution at the sample plane. The locally generated third-harmonic signal gives rise to interference patterns recorded on a visible detection camera, corresponding to the reciprocal lattice of the imposed intensity profile. Representative results include both square and hexagonal lattice configurations (see inset). The seemingly irregular pattern of the diffractive element shown in the schematic corresponds to the actual mask used to generate the square lattice and is obtained via standard inverse Fourier design

To experimentally validate this capability, we implemented a single-beam configuration in which the pump field is first shaped by a diffractive element before interacting with the AZO film. Due to the availability of components, diffractive masks were employed; however, the approach is fully general and directly transferable to other wavefront-shaping platforms. The diffractive element is designed to produce a prescribed two-dimensional intensity distribution at the position of the nonlinear layer (*d* ≫ *λ*), which in turn defines the spatial distribution of the locally generated third-harmonic signal. Representative results are shown in Fig. 6, where both square and hexagonal intensity profiles are imposed on the film, and the third-harmonic diffraction patterns recorded at the detection camera reproduce the corresponding reciprocal lattices.

These results demonstrate that the proposed system is not limited to interference-based gratings, but can support arbitrary and reconfigurable diffraction landscapes defined by the input wavefront. As such, the spatio-temporal modulation mechanism can be combined with standard wavefront-shaping techniques to extend the functionality of the platform beyond static or multi-beam implementations. For instance, low-index nonlinear films could be used in conjunction with spatial light modulators to considerably enhance their effective spatial resolution through frequency conversion.

## Discussion

In this work we realized a spatio-temporal photonic lattice by combining a physical nanostructure with an optically induced and fully reversible counterpart on a time-varying low-index platform. We selected a grating geometry because of its fundamental role and broad versatility in optics and photonics. To isolate scattering contributions arising from different origins, the permanent structural grating and the optically induced transient gratings are oriented orthogonally, so that their interference signatures separate clearly in the far field. By leveraging the large low-index nonlinearities, the use of an external probe is not strictly required since the system self-produces a short-wavelength probe via THG which is able to resolve nanoscale features. This yields a complete x-y interference map at the detector, in which structural and temporal interference orders appear with comparable intensities. The grating device also enables a substantial increase in THG compared with flat films due to local field enhancement.

In addition, the temporal lattice has also been interrogated using an independent external probe, enabling a clear separation between lattice formation and probing dynamics. This configuration provides direct access to the scattering response of the transient structure and reveals a substantially enhanced temporal tunability, with spectral shifts exceeding the intrinsic bandwidth of the probe.

Beyond the static, fabricated pattern, the non-stationary nature of our metasurface opens a multi-dimensional design space. The spatio-temporal meta-lattice does not need any lithographic step but is dynamically modulated by means of the pump wavelength, angles of incidence, and temporal delay between pulse trains. Tuning these knobs reshapes the effective 2D-meta-cell while also tuning the self-generated probe signal, producing large changes in amplitude, phase, spectrum, and momentum of the scattered light. Furthermore, by using phase masks we demonstrate that the approach extends beyond multi-beam interference. In this sense by using nonlinear single-beam ultrafast wavefront shaping we enable the generation of complex and programmable diffraction landscapes.

Our experiments exploit highly nonlinear, low-index conductive oxides to realize metagrating with exceptional ultrafast reconfigurability. Unlike purely index-modulation studies, we focus on optically induced scattering, a direct signature of light matter interaction that permits quantitative benchmarking of transparent conductors for temporal lithography. This approach moves device design from fixed fabrication constraints toward radiation engineering. This real time tunability on femtosecond timescales suggests new routes to ultrafast reconfigurable metasurfaces, adaptive spectral and spatial multiplexers, and engineered multi-channel high-harmonic sources.

## Methods

**Fig. 7:**
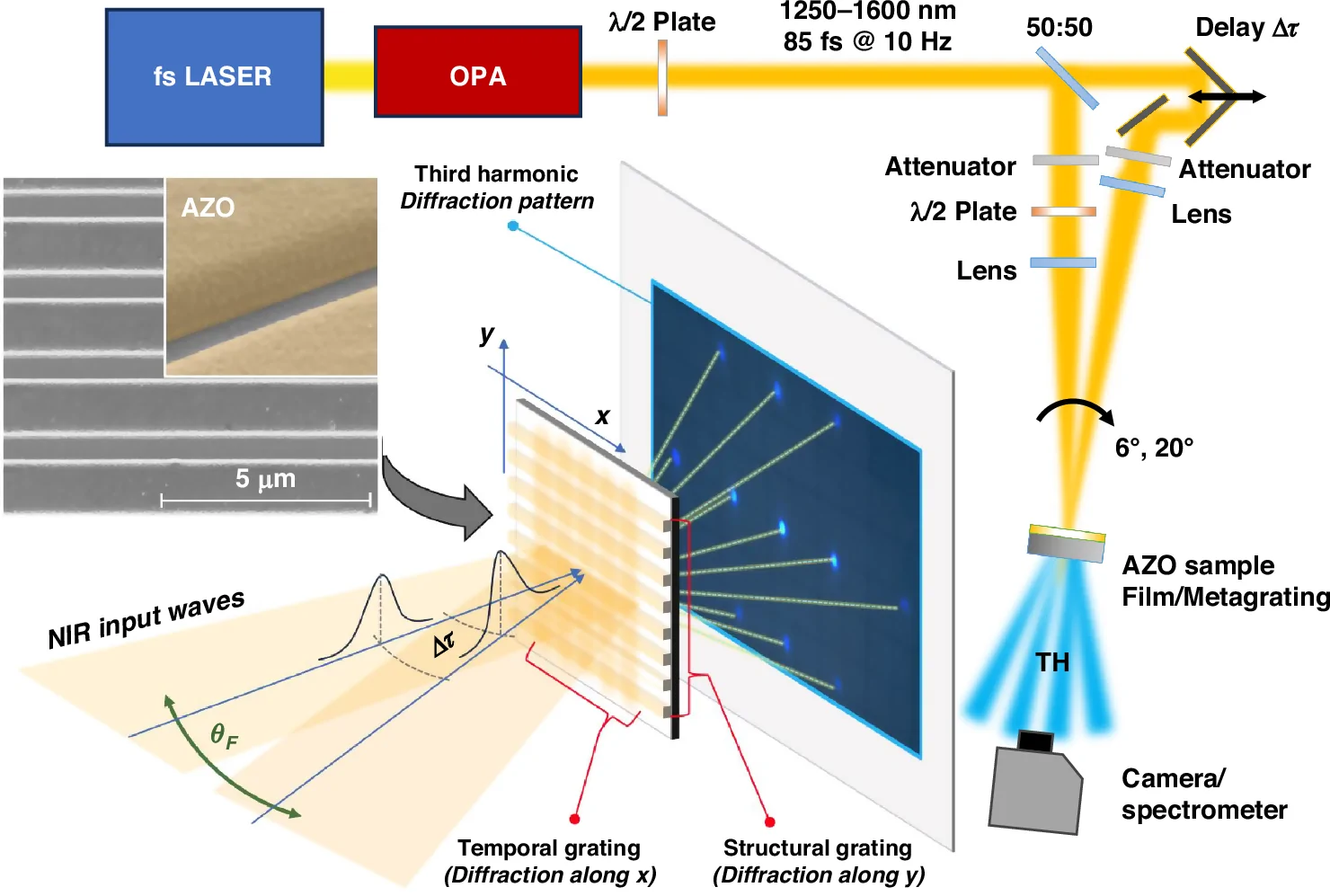
**Experimental set-up.** Experimental set-up and magnified schematic of the meta-grating sample, inclusive of an SEM image of the metagrating. Two NIR pump beams interact with a vertically oriented ridge grating, physically patterned onto a thin film of AZO. The system is excited with NIR 85 fs pulses close to the crossover wavelength of the sample. Interference between the two pump beams generates a time varying horizontal grating, orthogonal to the static vertical structure. An example interference pattern, attained by far eld recording of the internally generated TH signal, is also shown

Experiments were conducted using the setup reported in Fig. 7. Here, a femtosecond laser emitting ultrafast pulses in the wavelength range 1250–1600 nm, operates at 10 Hz. These 85 fs pulses are split into equal-intensity beams along separate propagation arms, one incorporating a variable delay stage.

The two NIR beams are then focused and spatially overlapped on the sample surface. This generates a TH signal with sufficient wavelength resolution to produce a double interference pattern in the far field which is due to both the static grating along the y-axis, and the optically induced transient grating along the x-axis. Experiments were conducted at different incident angles *θ*_i_ (6^∘^ and 20^∘^) using equal pump intensities in both arms. Small angles were chosen due to the limited device size and to optimize coupling into the system. All beams were p-polarized (i.e., electric field lying in the plane of incidence). Measurements were conducted in both single- and double-beam configurations on both a plain 500 nm-thick AZO film and a 1D fully etched metagrating (metalattice) with a groove density of  ≈ 670 lines ⋅ mm^−1^ and a duty cycle of 0.6. Nanopatterning was attained via Focused Ion Beam lithography (see Supplementary Information for details). Figure 7 inset shows a Scanning Electron Microscopy (SEM) image of the fabricated sample.

## Supplementary information

Supplementary Information

## Data Availability

The data that support the findings of this study are openly available from the Heriot-Watt Research Portal under a Creative Commons Attribution (CC BY) licence at https://doi.org/10.17861/1821a5a2-ad50-4b06-a006-c02894c76da2. Additional information can be made available from the corresponding author upon reasonable request.
