# Supplementary material for: Spatio-temporal photonic metalattice
Source: Light Sci Appl. 2026 Oct 29;15:392. doi: 10.1038/s41377-026-02452-w (PMC13720116; doi:10.1038/s41377-026-02452-w)
Supplement: Supplementary file 1 [file 41377_2026_2452_MOESM1_ESM.pdf]

Spatio-Temporal Photonic Metalattice
Supplementary Information

Sven Stengel[1†], Wallace Jaffray[1†], Farhan Ali[2], Mustafa Goksu Ozlu[3], Colton B. Fruhling[3], Maria Antonietta Vincenti[4,5], Domenico De Ceglia[4,5], Michael Scalora[1], Alexandra Boltasseva[3], Vladimir M. Shalaev[3], Andrea Di Falco[2], Marcello Ferrera[1*]

[1]Institute of Photonics and Quantum Sciences, Heriot-Watt University, Edinburgh, EH14 4AS, UK

[2]School of Physics and Astronomy, University of St. Andrews, St Andrews, UK

[3]Elmore Family School of Electrical Computer Engineering and Birck Nanotechnology Center, Purdue University, West Lafayette, Indiana, USA

[4]Department of Information Engineering,University of Brescia,Brescia, Italy

[5]CNR - National Institute of Optics, Via Branze 45, 25123 Brescia, Italy

*Corresponding author. Email: m.ferrera@hw.ac.uk

†These authors contributed equally to this work.

Appendix A   Materials and Methods

Appendix A.1   Fabrication details

The sample consisted of a 500 nm thick AZO film patterned into a periodic lattice structure and deposited on fused silica via pulsed laser deposition under low-oxygen conditions. An area of 200 µm × 200 µm was patterned with a one-dimensional grating with periodicity of about 1440 nm and with a duty cycle of 0.6, using a FEI Scios dual-beam scanning electron microscope (SEM) and focused ion beam (FIB). To avoid Ga ion implantation in the surrounding material and minimize electrostatic charging, the sample was coated with a 100 nm thick layer of Polymethylmethacrylate (PMMA) followed by thermal evaporation of a 10 nm thick carbon layer. These layers were subsequently removed by immersion in acetone in a gentle ultrasound bath. The PMMA was prepared via spinning at 1000 rpm for 60 sec, followed by backing for 60 s at 120 °C. The patterns were written after calibrating the exposure to guarantee the full etching of the AZO layer. After the exposure, the sample was immersed in acetone in a gentle ultrasound bath to remove the PMMA/Carbon layer.

Appendix A.2 Experimental configuration

The pump beam was supplied by an optical parametric amplifier (OPA) tunable within the near infrared (NIR) spectral region generating 100 fs pulses. These pulses were then split via a beamsplitter and then focused onto the sample with a $1/e^2$ spot size of 100 µm, resulting in peak intensities of approximately 300 GW cm^{-2} each. The pump was polarized linearly within the plane of incidence. Experimental results measuring the scattered third harmonic were taken with the pump beams arriving at angles of 6° and 20° to the sample's normal. For 6°, this spatial third harmonic data was combined with photodiode measurements to find the absolute third harmonic efficiency for several key configurations. Then, the time delay between the two arriving pumps was varied, and the spectrum of the generated third harmonic was recorded. For the 20° configuration, the pulse's central wavelength was scanned through 1320 nm and 1500 nm, and the spatial distribution of the scattered third harmonic light was measured.

Appendix B Theory

Appendix B.1 Momentum conservation in subwavelength films

Let the film normal be along $\hat{z}$ and the in-plane direction be $\hat{x}$. Two fundamental beams hit the film at the same internal angle θ_F on opposite sides of the normal, so their wavevectors are

$$\mathbf{k}_F^{(R)} = k_F\big(\sin\theta_F\,\hat{x} + \cos\theta_F\,\hat{z}\big), \qquad \mathbf{k}_F^{(L)} = k_F\big(-\sin\theta_F\,\hat{x} + \cos\theta_F\,\hat{z}\big). \tag{1}$$

Where k_F is defined as $2\pi/\lambda$ and λ is the incident pump wavelength. Suppose the third-harmonic photon is generated from n_r photons taken from the right beam and n_l photons from the left beam ($n_r + n_l = 3$). The TH direction follows the vector sum of the contributing fundamentals:

$$\mathbf{K} = n_r\mathbf{k}_F^{(R)} + n_l\mathbf{k}_F^{(L)}. \tag{2}$$

Decomposing into components:

$$K_x = (n_l - n_r)k_F\sin\theta_F, \qquad K_z = (n_l + n_r)k_F\cos\theta_F. \tag{3}$$

Thus, the third harmonic emission angle θ_{TH} satisfies

$$\tan\theta_{TH} = \frac{K_x}{K_z} = \frac{(n_l - n_r)k_F\sin\theta_F}{(n_r + n_l)k_F\cos\theta_F} = \frac{n_l - n_r}{n_l + n_r}\,\tan\theta_F. \tag{4}$$

Hence,

$$\theta_{TH} = \arctan\left(\frac{n_l - n_r}{n_l + n_r}\,\tan(\theta_F)\right) \tag{5}$$

Higher order mixing processes can however produce TH emission outside of the fundamental cone. For example, if $n_r = 5$ (fifth harmonic) and $n_l = -2$ (two conjugate fundamental photons), then $n_r + n_l = 3$. Thus, it is possible to generate a third harmonic photon with $\theta_{TH} = \frac{7}{3}\theta_F$ (for small

θ_F). That said, the efficiency of this process is much smaller when compared to the direct third harmonic generation as it relies on collinear fifth or seventh harmonic generation. Additionally, the anomalous high angle third harmonics do not line up with the high order wave mixing processes, so we can safely discount this possibility.

Appendix B.2 Transient grating parameter estimation

The outer third harmonics ($m = -1$), allow us directly probe the transient grating and to confirm its existence and pitch (see main manuscript Fig. 2a-4). Beyond this, we can estimate a few key parameters about the transient grating. To provide a general idea on the strength of the spatial refractive index modulation, in Fig. S1 top panels, we plot the intensity at the surface of our sample as a function of position for both 6° and 20° incidence angles. Using the calculated intensities from the two-beams geometry we then estimate the associated spatial variations in real and imaginary index for the 6° (bottom left) and 20° (bottom right) input beam angles. This calculation demonstrates that our transient grating has a real index modulation of about 0.5.

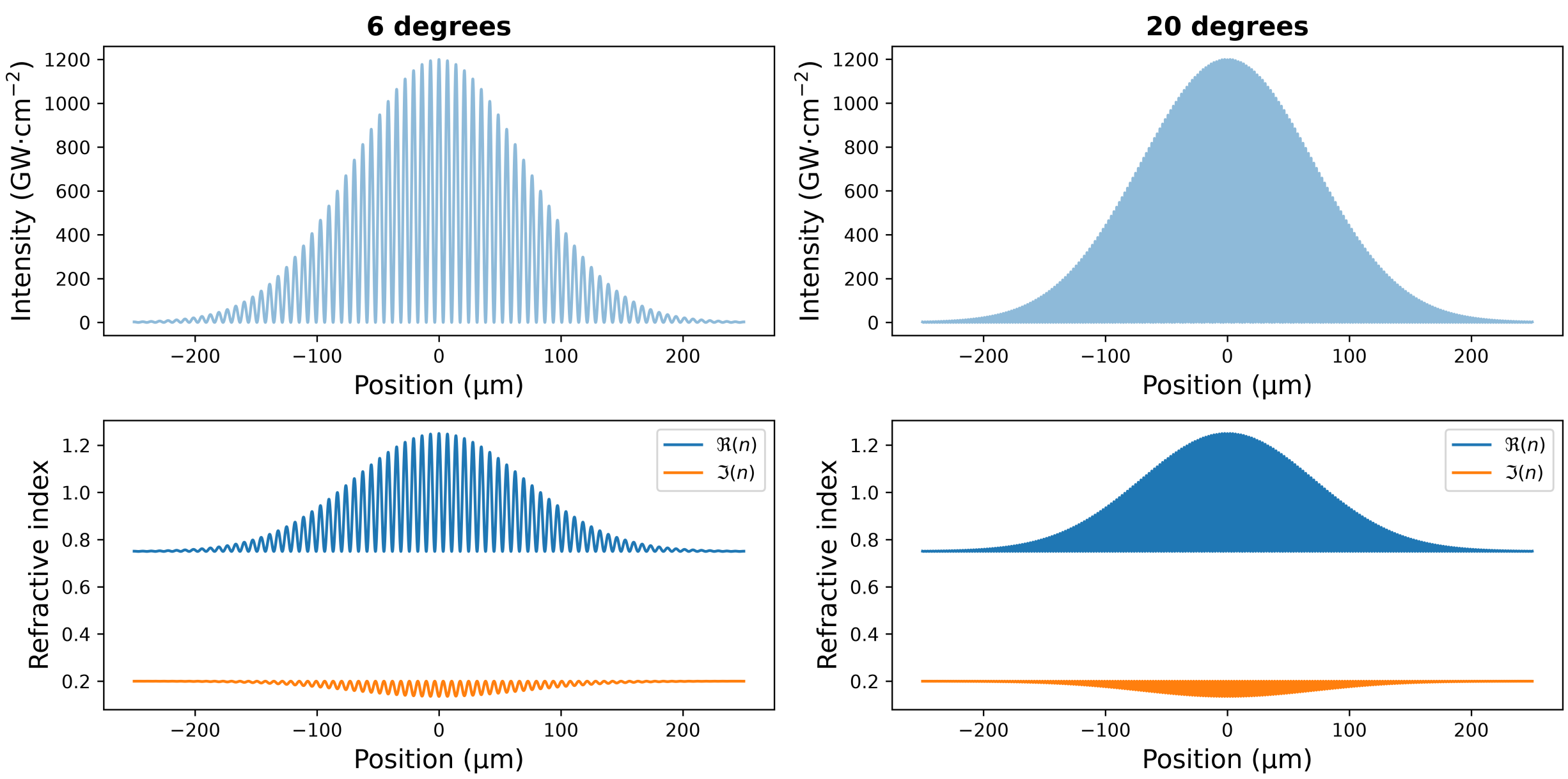

Figure S1: Transient grating additional analysis. Top panels report dual beam surface intensities as a function of position for 6° (left) and 20° (right). Bottom panels report the estimated associated spatial index variations.

Appendix B.3 Grating third harmonic enhancement

Near the ENZ condition, the real part of the permittivity of AZO approaches zero so that the refractive index becomes smaller than both the superstrate (air) and substrate (glass) of the grating. This scenario creates a large impedance mismatch with the surrounding environment, enabling

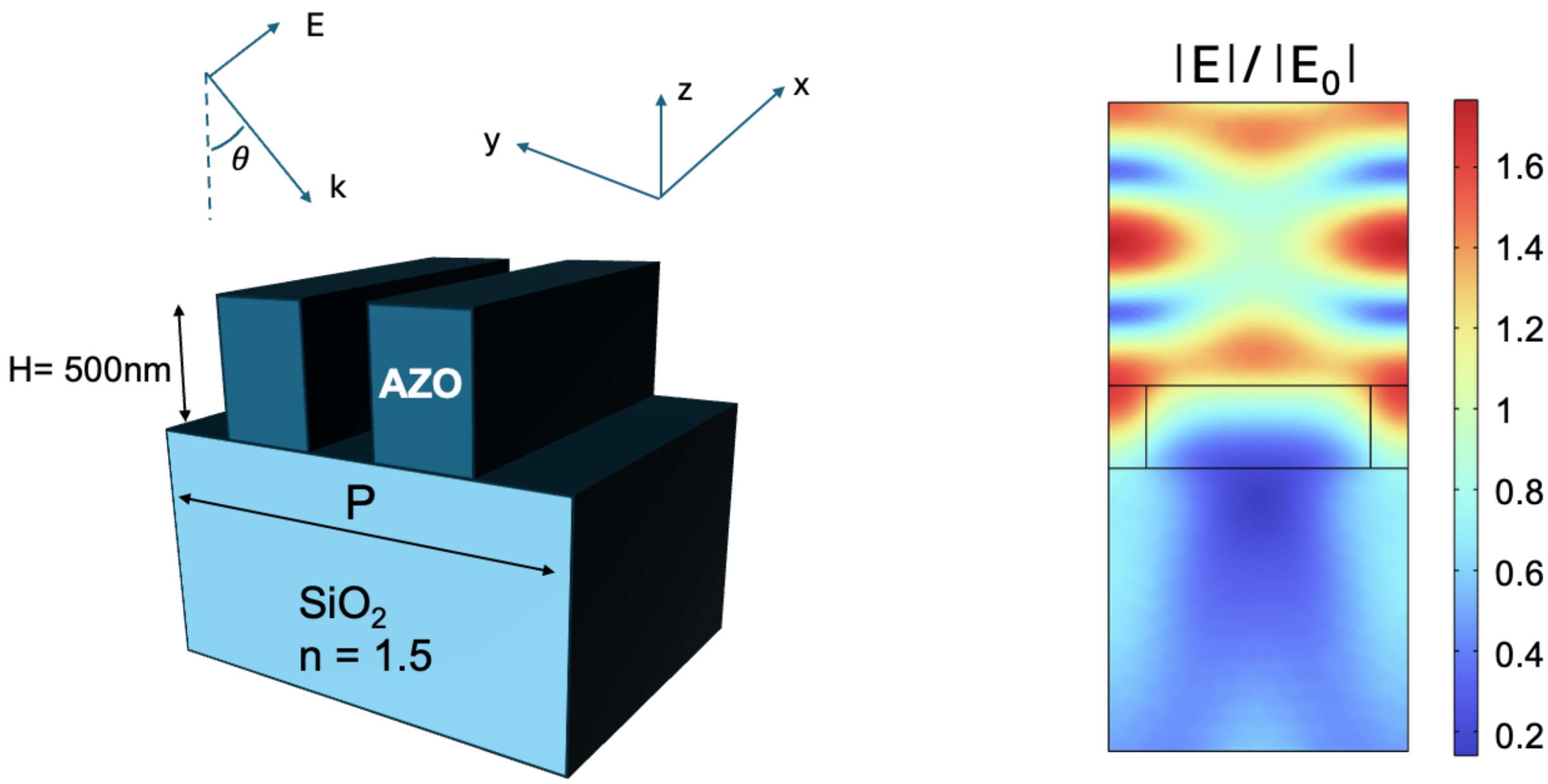

Figure S2: Grating induced local field enhancement. Left panel shows general grating structure, design parameters, and input beam k-vector. Beam input angle is set to 6°, grating pitch to 1670 nm, and filling fraction to 0.5. Right panel shows local electric field amplitude E, normalized to the amplitude of the incident electric field amplitude E_0.

strong field compression at interfaces and within the subwavelength gaps of the periodic structure. This in turn leads to the formation of hybrid gap–plasmon resonances, in which the electromagnetic field is strongly confined within the grating grooves or trenches, with characteristics analogous to classical metal–insulator–metal gap plasmons [S1-S3]. These modes, which arise from the interplay of subwavelength geometry, extremely low permittivity values, and momentum-matching conditions imposed by the grating, still manifest as intense localized fields within the gap region as in metallic gratings, while acquiring some unique characteristics given by the ENZ condition [S4, S5]. This allows the fields to penetrate the material more than in traditional gap-plasmons. It is also worth mentioning that the requirement for an angled incidence to produce the temporal grating also leads to a further increase of the generated third harmonic signal which is due to the enhancement of the longitudinal field component at the interface with an epsilon near zero material. This is supported by the local field enhancement distribution reported on the right side of Fig. S2 [S6, S7].

For the grating period employed in the experiments, $P = 1440$ nm, we also compare the simulated spectral dependence of the THG efficiency with the corresponding experimental measurements, as shown in the left panel of Fig. S3. The agreement between theory and experiment confirms that the numerical model captures the main features of the measured nonlinear response. This comparison validates the use of the selected grating geometry for the subsequent spatio-temporal modulation experiments and further indicates that additional geometrical optimization is not critical for the physical interpretation of the results.

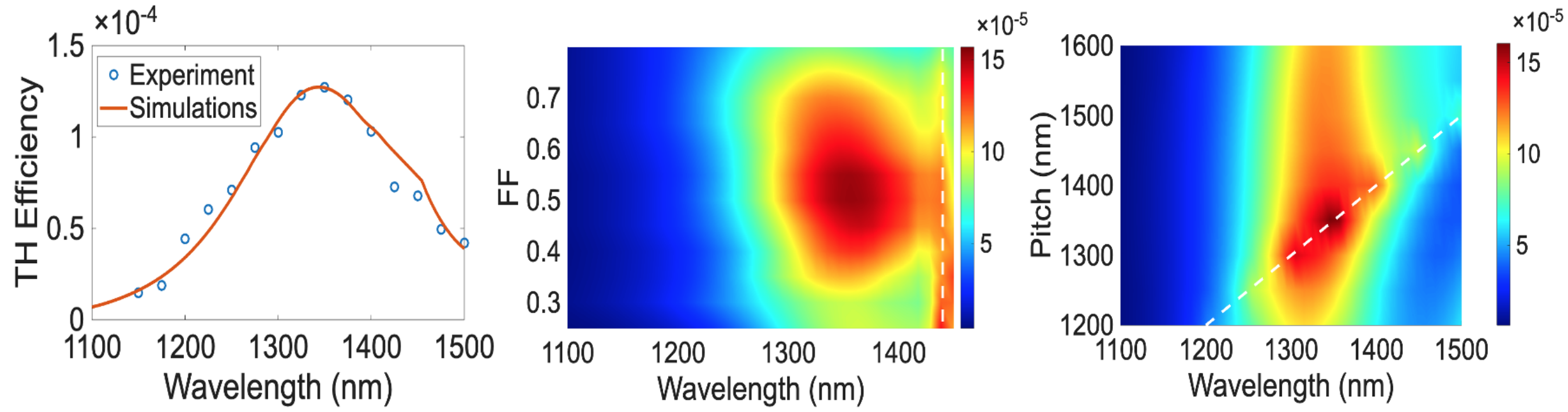

Figure S3: THG parameter scan. Left panel shows THG efficiency as a function of pump wavelength. Experimental data (blue circles) are fit with our material model (red line). Central panel shows parameter scan of conversion efficiency versus filling fraction (FF). Right panel shows parameter scan of conversion efficiency versus grating pitch for FF=0.5 (Maximum efficiency in middle panel).

To investigate the influence of the grating geometry on the THG emission, we performed nonlinear numerical simulations by varying both the filling fraction (see Fig. S3 middle panel) and the grating period (see Fig. S3 right panel). In the latter case we the filling fraction is fixed at FF = 0.5, corresponding to the optimal geometry which also closely aligns with the experimental devices. The simulated THG efficiency is reported on the right-hand side of Fig. S3 as a function of both pump wavelength and grating period for an incident intensity of 600 $\mathrm{GW} \cdot \mathrm{cm}^{-2}$.

The calculated maps show that variations of the grating period have only a limited impact on the overall THG efficiency within the investigated parameter range. This indicates that the nonlinear response is not critically dependent on a narrowly optimized grating geometry but is primarily governed by the material properties associated with the ENZ region. The white dashed lines in Fig. S3 marks the Rayleigh–Wood anomaly corresponding to the onset of the first diffraction order. A moderate enhancement of the THG signal is observed in proximity to this condition, consistent with the field localization expected near the diffraction threshold. Nevertheless, the overall spectral response remains largely determined by the intrinsic nonlinear properties of the material, while the grating geometry provides only a secondary contribution.

In the context of the present work, the TH conversion efficiency must be interpreted within the appropriate physical regime. In fact, despite operating over a deeply subwavelength thickness (hundreds of nanometres), without phase matching or resonant enhancement, the measured TH efficiency still remains comparable to values reported in substantially more favorable experimental settings. This is primarily enabled by the exceptionally large nonlinearities exhibited by low-index transparent conducting oxides.

References

S1. F. Ding, Y. Yang, R. A. Deshpande, and S. I. Bozhevolnyi, "A review of gap-surface plasmon metasurfaces: fundamentals and applications," Nanophotonics, 7(6), 1129–1156 (2018).

S2. H. Choo, M.-K. Kim, M. Staffaroni, T. J. Seok, J. Bokor, S. Cabrini, P. J. Schuck, M. C. Wu and E. Yablonovitch, "Nanofocusing in a metal–insulator–metal gap plasmon waveguide with a three-dimensional linear taper," Nature Photonics, 6, 838–844 (2012).

S3. M. G. Nielsen, A. Pors, O. Albrektsen, and S. I. Bozhevolnyi, "Continuous layer gap plasmon resonators," Optical Express 19 (20), 19310–19322 (2011).

S4. R. G. Bikbaev, S. Ya. Vetrov, and I. V. Timofeev, "Transparent conductive oxides for the epsilon-near-zero Tamm plasmon polaritons," J. Opt. Soc. Am. B 36, 2817–2823 (2019).

S5. S. Choudhary, J. Patrow, A. Pennathurs, H. Song, "Strongly Coupled Plasmon Polaritons in Gold and Epsilon-Near-Zero Bifilms," ACS Photonics 10 (1), 162–169 (2023).

S6. A. Capretti, Y. Wang, N. Engheta, and L. Dal Negro. "Enhanced third-harmonic generation in Si-compatible epsilon-near-zero indium tin oxide nanolayers." Optics letters, 40(7), 1500-1503 (2018).

S7. T. Luk, D. De Ceglia, S. Liu, G. A. Keeler, R. P. Prasankumar, M. A. Vincenti, M. Scalora, M. B. Sinclairand, S. Campione. "Enhanced third harmonic generation from the epsilon-near-zero modes of ultrathin films," Applied Physics Letters, 106(15). (2015).
